# Biosensing Technologies: A Focus Review on Recent Advancements in Surface Plasmon Coupled Emission

**DOI:** 10.3390/mi14030574

**Published:** 2023-02-28

**Authors:** Seemesh Bhaskar

**Affiliations:** 1 Nick Holonyak Jr. Micro and Nanotechnology Laboratory (HMNTL), University of Illinois at Urbana-Champaign, Urbana, IL 61801, USA; seemeshb@illinois.edu; 2 Carl R. Woese Institute for Genomic Biology, University of Illinois at Urbana-Champaign, Urbana, IL 61801, USA; 3 Department of Electrical and Computer Engineering, University of Illinois at Urbana-Champaign, Urbana, IL 61801, USA

**Keywords:** surface plasmon coupled emission, luminescence, nano-engineering, ferroplasmon, cryosoret, photonic crystal-coupled emission, smartphone diagnostics

## Abstract

In the past decade, novel nano-engineering protocols have been actively synergized with fluorescence spectroscopic techniques to yield higher intensity from radiating dipoles, through the process termed plasmon-enhanced fluorescence (PEF). Consequently, the limit of detection of analytes of interest has been dramatically improvised on account of higher sensitivity rendered by augmented fluorescence signals. Recently, metallic thin films sustaining surface plasmon polaritons (SPPs) have been creatively hybridized with such PEF platforms to realize a substantial upsurge in the global collection efficiency in a judicious technology termed surface plasmon-coupled emission (SPCE). While the process parameters and conditions to realize optimum coupling efficiency between the radiating dipoles and the plasmon polaritons in SPCE framework have been extensively discussed, the utility of disruptive nano-engineering over the SPCE platform and analogous interfaces such as ‘ferroplasmon-on-mirror (FPoM)’ as well as an alternative technology termed ‘photonic crystal-coupled emission (PCCE)’ have been seldom reviewed. In light of these observations, in this focus review, the myriad nano-engineering protocols developed over the SPCE, FPoM and PCCE platform are succinctly captured, presenting an emphasis on the recently developed cryosoret nano-assembly technology for photo-plasmonic hotspot generation (first to fourth). These technologies and associated sensing platforms are expected to ameliorate the current biosensing modalities with better understanding of the biophysicochemical processes and related outcomes at advanced micro-nano-interfaces. This review is hence envisaged to present a broad overview of the latest developments in SPCE substrate design and development for interdisciplinary applications that are of relevance in environmental as well as biological heath monitoring.

## 1. Introduction

Fluorescence spectroscopy has revealed great promise with myriad probes and devices demonstrating a rich spectrum of applications related to biological and chemical sensing, topographical analysis, immunoassays, optofluidics, forensics, microscopy, single molecule detection, environmental health monitoring as well as myriad point-of-care (POC) diagnostic technologies [1,2,3,4]. In an attempt to obtain enhanced signal intensities in traditional fluorescence-based analytical detection methodologies, it has been synergized with the metallic/plasmonic nanomaterials garnering active optoelectronic functionalities [5,6,7,8,9,10]. Such explorations have significantly advanced the frontier areas of biosensing research with several economical and industrial applications. This is on account of the ability of researchers to tailor the excitation and emission intensities of fluorescent moieties by placing them in the proximal vicinity of the plasmonic nanoparticles (NPs) sustaining localized surface plasmon resonances (LSPR) [10,11,12,13]. The high-gradient electromagnetic (EM) field intensity provided by such LSPRs assist augmented sensitivity in analyte detection on account of substantial modification in the local density of states (LDoS) [10,14,15,16]. Moreover, it has been observed that the resonant charge density perturbations in plasmonic NPs interact with the fluorophores in the near-field, and consequently, the emitters assist in the generation of plasmons that radiate into the far-field, carrying the emission characteristics of the fluorophores [10,17]. From this perspective, the resulting hybrid system of metal-fluorophore generates an efficient plasmophore (plasmon + fluorophore), transmitting the optical features of the individual counterparts. Furthermore, such an increase in the fluorescence intensity is attributed to the high radiative decay rate, robust photostability as well as the decrease in the lifetimes, ensuing an associated upsurge in the global quantum yield. Such investigations where the light (emission)–matter (nanomaterial) interactions assist in optical trapping, tuning, control, evaluation and manipulation of the resultant fluorescence intensity have developed into a mature field termed ‘plasmon-enhanced fluorescence (PEF)’ [8,10,18,19,20]. These explorations have supported the comprehension of diverse novel phenomena in the sub-fields of nanophotonics, such as metal-dependent plasmonics [3], graphene-based plasmonics [21], dielectric-dependent metamaterials [22] and photonic crystals (PCs) [23,24], to name a few.

Nevertheless, in spite of the abovementioned application potential of PEF technologies, the far-reaching capabilities of the fluorescence-based analytical detection systems are compromised on account of the omnidirectional (isotropic) emission and allied low-signal collection efficiency (<1%), photobleaching and high background noise [8,10,17]. In order to overcome these limitations, Lakowicz and co-workers developed an innovative technology termed surface plasmon coupled emission (SPCE) in a series of research credentials termed radiative decay engineering, ‘one to eight’ [17,25,26,27,28,29,30,31]. SPCE platform is a prism coupling technique where the fluorescence is coupled to the surface plasmon polaritons (SPPs) of the metal thin film assisting in the realization of >50% signal collection efficiency, on account of exceptional directionality of emission. Further to the high p-polarized attribute of the emission signal (reinforced by the SPPs of the metal thin film), the SPCE fosters a 10–15-fold enhancement in the signal vis-à-vis conventional fluorescence, with high background suppression and spectral resolution [28]. In an attempt to further increase the fluorescence enhancements observed in the SPCE framework, Chowdhury et al. demonstrated the utility of plasmonic AgNPs as active spacer material [32]. This has assisted in the realization of 60-fold SPCE enhancements; following which, several other nano-architectures with numerous sizes, shapes and assemblies have been examined in the SPCE platform for achieving amplified SPCE enhancements [33,34,35,36,37,38,39,40]. Such synergy of fluorescence spectroscopy and applied nano-research with effective nano-engineering strategies has advanced the spectro-plasmonic modalities in the SPCE platform with newer applications and processes including, but not limited to: ultra-high sensitivity [41,42,43], CNT-assisted augmented coupling [44], cardiovascular disease and food biomarker monitoring [45], fluorescent polymer brushes for large angle studies [46], interfacial molecular beacon-related explorations [47], cavity-void plasmon coupling in nano-assemblies sustaining Bragg and Mie plasmons [48], adsorption-desorption analysis [49], lightning-rod effect [50], graphene π-plasmon hybrid coupling [51,52,53,54], mesoporous carbon florets for photon cascading in nanocavity [55], lower-to-higher aggregates coupling [56], magneto-plasmonics [57], PLEDs [58], simultaneous multianalyte sensing [59] and other cost-effective biosensing applications [60,61,62,63,64,65,66]. In spite of these developments, the three major long-standing limitations of the SPCE technology development have been: (i) a moderate increase in the SPCE signal intensity [67,68,69]; (ii) the restricted coupling of excited fluorophores to the s-polarized modes of the metal-dielectric plasmonic waveguides [70,71,72]; (iii) high Ohmic losses sustained by the SPCE platform [73,74,75]. In order to address these limitations of the SPCE platform, Ramamurthy and co-workers, developed strategies with careful considerations of nano-engineering, photo-plasmonics and materials chemistry approaches [76,77,78,79,80,81]. Following a brief discussion on the state-of-the-art capabilities of the SPCE platform in Section 2, this focus review provides a systematic understanding of the different approaches adopted to circumvent the abovementioned enduring drawbacks in SPCE. In order to address the first limitation (discussed in detail in Section 3), a novel cryosoret nano-engineering technology was developed in a spotlight article with the intriguing title ‘Welcome to Nano 4.0′ [54]. Here, the importance of exploring precise nanoparticle assemblies is discussed, highlighting the importance of investigating delocalized Bragg and localized Mie plasmons, in order to achieve unprecedented SPCE enhancements. The ability of nano-assemblies to overcome conventionally observed quenching phenomena is detailed. In order to circumvent the second limitation (deliberated in detail in the penultimate Section 4), a new platform based on the ferromagnetic-plasmonic hybrid nano-engineering of NPs over the SPCE interfaces is demonstrated with the development of ferroplasmon-on-mirror (FPoM) technology (which is analogous to the well-known nanoparticle-on-mirror (NPoM) platform) [71]. Finally, in order to overcome the third limitation (deliberated in detail in the final Section 5), an alternate platform that completely eliminates the usage of lossy plasmonic surfaces is demonstrated with the alternating high and low refractive index nanolayers in a photonic crystal framework, engendering the photonic crystal-coupled emission (PCCE) platform [11,40,42]. Here, the inevitable shortcomings of the SPCE platform, such as surface oxidation and high Ohmic losses, are addressed. Furthermore, a separate section (Section 6) is devoted to highlight the futuristic scope and research opportunities in the broad arena of SPCE technology development, as well as the utility of cryosorets and ferroplasmonic nanomaterials for interdisciplinary applications. Furthermore, a section is dedicated to discussing the challenges and the limitations of the developed techniques, thereby emphasizing the need for further research in this direction. In summary, this focused editorial review is envisaged to ameliorate the existing biosensing methods with the advent of novel technologies pertaining to cryosorets, FPoM and PCCE platforms. In light of these observations, the emphases are on the recent advancements in SPCE technology development, thereby assisting researchers, academicians and industrial experts to employ the latest know-how with respect to fluorescence-based detection modalities for environmental and biological sensing devices.

## 2. Surface Plasmon Coupled Emission (SPCE) Technology

**Figure 1:**
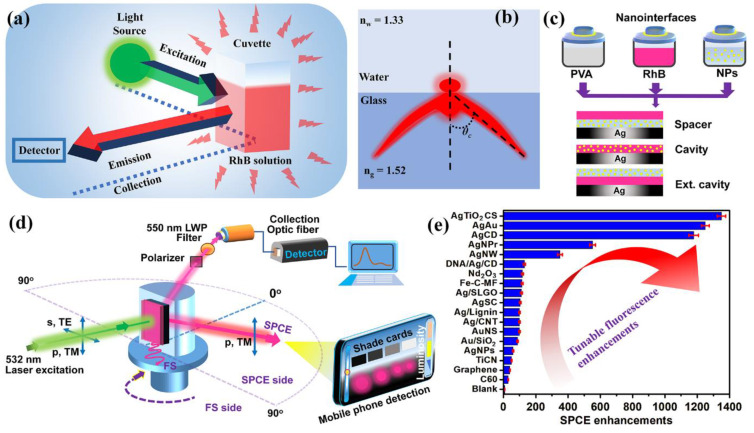
Conceptual schematic of (**a**) fluorescence emission (of RhB) recorded by conventional fluorescence spectrophotometer, (**b**) the angular dispersion of fluorescence emission as observed in the water-glass interface. The angle shown is critical angle (*θ*_C_) of emission. Adapted with permission from [11]. (**c**) Schematic of the spacer, cavity and ext. cavity nanointerfaces. (**d**) Optical setup used for SPCE experimental work with reverse Kretschmann (RK) configuration. The detection system is carried out using the conventional Ocean Optics detector, as well as the mobile phone-based detection system. Adapted with permission from [64]. (**e**) Tunable enhancements in the fluorescence enhancements observed using different nanomaterials and nanohybrids in SPCE platform. (Acronyms: AgTiO_2_ CS: silver titanium dioxide cryosoret; AgAu: silver-gold nanohybrid; AgCD: silver NP decorated-carbon dots; AgNPrs: silver nanoprisms; AgNW: silver nanowire; DNA/Ag/CD: DNA based AgCD composite; Nd_2_O_3_: neodymium (III) oxide; Ag/SLGO: silver NPs decorated on single layer graphene oxide; Ag/lignin: lignin-based AgNPs; Ag/CNT: carbon nanotubes decorated with AgNPs; AuNS: gold nanostars; Au/SiO_2_: gold NPs decorated on silica NPs; TiCN: titanium carbonitride; C60: carbon allotrope or buckminsterfullerene, (C60-Ih) [5, 6] fullerene).

Following the pioneering work by Lakowicz and co-workers, SPCE technology has been implemented in the advancement of several biosensing platforms [82,83,84]. This section provides a brief overview of the SPCE platform and the associated nanointerfaces. Figure 1 showcases a typical configuration in which the fluorescence is captured using a cuvette in a conventional fluorescence spectrophotometer. Traditionally, the detectors are placed at 90° in order to avoid the direct light from the irradiation source, as well as any other interference [8,10,11]. In this regard, as the detector is placed in a fixed location at one particular angle, the collection efficiency is drastically lowered as the light emitted is isotropic from the cuvette. Furthermore, conventional fluorescence spectroscopy has several drawbacks: (i) low signal collection efficiency; (ii) poor resolution of emission peaks; (iii) lower sensitivity; (iv) requirement of cumbersome equipment; (v) omnidirectional emission property with negligible recognition of low quantum yield emitters [8,10,11,17]. For the radiating dipoles placed at the glass-water interface, the emission in the relatively HRI region (glass, n_g_ = 1.52) develops into a partially directional emission (Figure 1b). This is due to the effect of critical angle (*θ*_C_), at which the evanescent field is generated at the interface, presenting an off-normal (non-isotropic) and partially directional and not polarized emission [10]. While these are the preliminary observations with regard to the emission, as discussed in detail elsewhere [10], the emission pattern can be channelized into sharply directional and polarized emissions using SPCE and PCCE platforms (Figure 1d).

The generally explored nanointerfaces in the SPCE platform are presented in Figure 1c, presenting the spacer, cavity and extended (ext.) cavity nanointerfaces, and the SPCE platform is schematically shown in Figure 1d [11,33,34,35,36]. The nanointerfaces are often fabricated using the spin-coating methodology, wherein the nanomaterial and the fluorophores of interest are doped in a polymer matrix and spin coated over the SPCE platform. The SPCE enhancements depend on several characteristics of the nanomaterials used, and also significantly depend on the nanointerfaces utilized. In the spacer nanointerface, in principle, the nanomaterial functions as an active spacer material between the radiating dipoles (fluorescent moieties) and the SPPs of the metallic thin film [61,64,67]. In the cavity nanointerface, the infinitesimal nanogaps generated between the nanomaterial and the metal thin film sustain plasmonic hotspots where the radiating dipoles are sandwiched [34,37,39]. Furthermore, as the name suggests, the cavity hotspots in the cavity nanointerface are extended to a defined distance in the ext. cavity interface [6]. 

While the spacer and ext. cavity nanointerfaces, as observed in Figure 1c, are constituted by two separate nanolayers, the cavity nanointerface is a single nanolayer. Consequently, the surface-induced quenching effects are significantly observed in the cavity nanointerface compared to the other two. By and large, the performance of these architectural designs has been examined with different nanomaterials and a comprehensive analysis of such explorations would demand the usage of meta-analysis and associated artificial intelligence and machine learning tools to comprehend the opto-electronic response of nanomaterials generated from a combination of elements from different parts of the periodic table [54,64,71]. In a typical SPCE experiment, the SPPs are generated by illumination at an appropriate angle, which can satisfy the phase matching conditions at the metallo-dielectric nanointerface [9,10,11]. The evanescent field is generated via both the Kretschmann-Raether (KR) and Reverse Kretschmann (RK) configurations, although the latter is more conducive for large-scale production and incorporation of the SPCE platform in biosensing approaches [9]. This is on account of the fundamental difference between the two technologies in terms of the laser excitation and emission collection attributes. While the excitation and emission are performed from the curved surface of the prism in the KR configuration, the excitation is carried out from the flat surface of the prism (or from the sample side) in the case of RK optical configuration [9,10,11]. In this review, the focus is deliberated on the RK configuration on account of its benefits, as well as to draw conclusive comparisons between the SPCE, as well as FPoM and PCCE technologies (discussed in subsequent sections). In a typical experiment, the nano-engineered SPCE substrate is affixed over the prism using an index matching fluid, as shown in Figure 1d. The prism is then mounted on a rotating stage and the emission is collected using appropriate optical filters and polarizers using an optic fiber. The final detection and the analysis of the SPCE emission signal is carried out using two detection systems: (i) the exorbitant Ocean Optics detector system; (ii) the cost-effective smartphone-based detection platform. This departure from conventional detection systems towards hand held devices has been recently pursued on account of the advantages of the latter in terms of easy transportability, unparalleled data acquisition ability, superior computing and ever-refining premium quality camera technologies [34,35,37,39].

In order to enhance the sensitivity of the detection devices, different nano-engineering techniques have been investigated and explored over the SPCE platform using myriad nanomaterials, including metallic nanomaterials (Ag, Au, Pt, Cu), dielectric nanomaterials (Nd_2_O_3_, SiO_2_, TiO_2_, TiC, TiN, TiCN), ferromagnetic nanomaterials (Fe_2_O_3_, Nd_2_O_3_-Ag or NdAg nanohybrids), homometallic and heterometallic, bi-, tri-, tetra-metallic nanohybrids, as well as graphene Dirac fermions and other 2-dimensional material-sustaining partially propagating plasmons, etc. [9,10,55,85,86,87,88,89,90,91]. The EM field intensity in the spatial regions of nanogaps between the NPs and the metallic thin film is dependent on several factors, such as the shape (rods, triangles, urchins, spheres, cubes stars, and wires), size (<10 nm, 10 nm–100 nm, >100 nm), architecture (core-shell, decorated), surface roughness, nature of adjacently situated plasmonic and/or dielectric NPs as well as the immediate environment and its refractive index [92,93,94,95,96,97]. Extensive theoretical analysis of the utility of such nanomaterials for efficient photo-plasmonic hotspot generation have been carried out using discrete dipole approximation (DDA) [10], finite-difference time-domain (FDTD) [9,48,71,74] and COMSOL Multiphysics simulations [34,98] to obtain a comprehensive understanding of the hotspot behavior. These explorations have assisted in the realization of new opto-electronic phenomena at nano-dimensions, such as Casimir force, Rabi splitting, Fabry-Perot photonic mode-coupling, Fano resonance, quantum confinement and the Purcell effect in the SPCE platform, rendering scientific insights into physicochemical interactions at advanced interfaces [9,11,55]. These research studies have resulted in the development of intriguing biosensing platforms, thereby supporting translational photonics research in addition to providing newer insights from the basic (simulations) and applied research perspectives.

## 3. Disruptive Nano-Engineering Using Cryosorets Technology in SPCE Interface

Disruptive nano-engineering has advanced the understanding of light-matter interactions in nano-regimes with newer perceptions of interfacial processes [99,100,101,102,103,104,105,106]. In the past decade, although different nanomaterials and nanohybrids have been extensively explored, as functional spacers, cavities and ext. cavity nanointerfacial materials in the SPCE platform, the utility of precise nano-assemblies for fluorescence enhancements has remained a daunting challenge on account of the complex procedures involved in obtaining such assemblies with high stability. Subramaniam and co-workers demonstrated the importance of exploring the soret nano-assemblies synthesized using a lower temperature (−18 °C) gradient for surface-enhanced Raman scattering and associated biosensing applications [107,108]. Following this revelation, in 2022, Ramamurthy and co-workers demonstrated the utility of extremely low temperatures (−80 °C, −150 °C and −196 °C) for nano-assembly synthesis via cryosoret nano-engineering (CSNE) generating metal, metal-dielectric and metal-graphene oxide-based hybrid nano-assemblies with tailorable plasmonic hotspots [54]. This section provides an executive summary and key highlights of CSNE for the realization of precise nano-assemblies and associated photo-plasmonic hotspots for applications in biosensing using the SPCE platform.

It is informative to provide a brief overview of the available nano-assembly routes and their importance in the broad arena of photo-plasmonics. Self-assembly is one of the several other different technologies available for combining materials with individually unique properties into a highly organized construct with collective functional properties [109]. Fundamentally, the self-assembly is a dynamic evolution occurring in a sample under consideration where the originally disordered elements of the sample existing in a particular equilibrium reoriented and re-organized into a well-ordered purposeful construct where the equilibrium states of the individual components reside in an energetic minimum [110,111,112,113]. By and large, such a methodology of obtaining the self-assembly of nanomaterials can be categorized in to the following: (i) a template-dependent route based on hard and soft templates, where an addition of external stimuli can essentially stimulate the self-assembly process. External stimuli can include a variety of processes such as solvent-driven, acid/base-driven, ion-driven, macromolecules-driven and redox-reaction-driven to name a few. (ii) The effective use of an electric field such as ac and dc (also termed as dielectrophoresis), as well as their alternating properties, (iii) static and dynamic magnetic fields, and (iv) light-driven self-assembly [110,111,112,113,114]. Several of these technologies have been explored worldwide for different applications ranging from chem-bio sensors to the Internet of Things (IoT). Based on the cogent groupings of the abovementioned technologies, the method implemented for nano-assembly synthesis has been broadly classified as (a) hierarchical, (b) directed and (c) coassembly [109,110,111]. While the routes available to prepare nano-assembly to date generally involve the use of directing and orienting groups, as well as different external stimuli conditions, the use of lower temperatures has been seldom discussed. Against this background, this section highlights the use of nano-assemblies obtained via subjecting the nanoparticle solutions to lower temperatures (−80 °C, −150 °C and −196 °C), such as for obtaining precise nano-assemblies with tailorable plasmonic hotspots [54]. 

**Figure 2:**
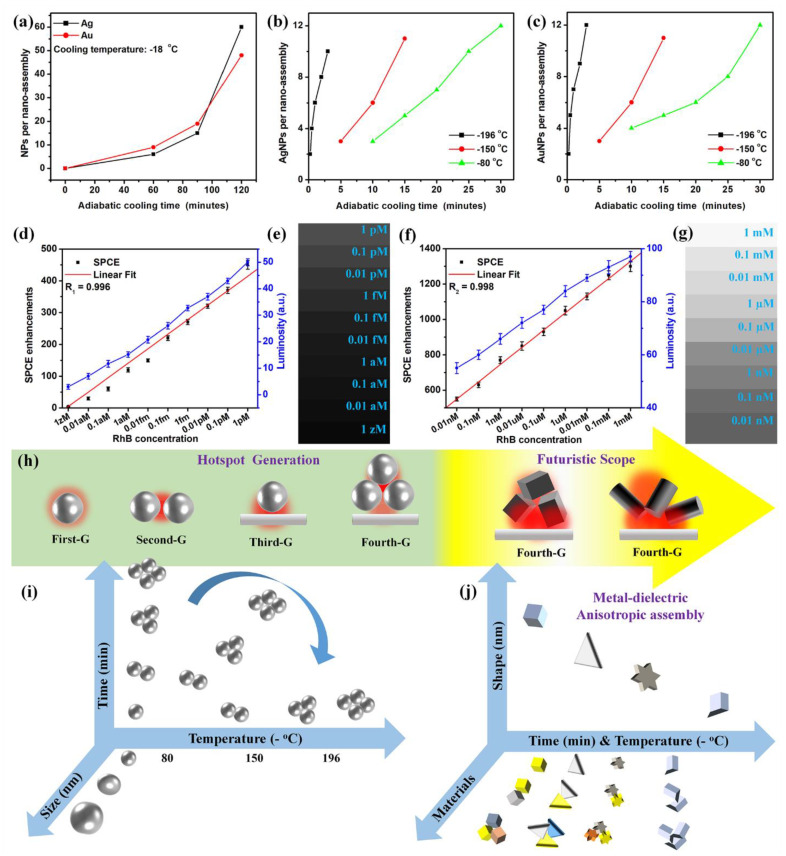
(**a**) AgNPs and AuNPs constituting a single assembly under −18 °C adiabatic cooling. (**b**) AgNPs constituting a single assembly under −80 °C, −150 °C and −196 °C adiabatic cooling. (**c**) AuNPs constituting a single under −80 °C, −150 °C and −196 °C adiabatic cooling. (**d**,**f**) Sensing of RhB presenting a single molecular limit of detection (zeptomolar). The corresponding luminosity values are presented in the right y-axis along with the respective shade cards shown in Figure (**e**,**g**), respectively. (**h**) The progress in diverse generations of hotspots in CSNE technology presenting opportunities for future explorations. (**i**,**j**) Snapshot of the plausible permutations and combinations of cryosoret-based nano-engineering available to the widespread community of nanotechnology and photo-plasmonics. Adapted with permission from [54].

It is informative to provide the details of the general mechanism of soret (nano-assembly) formation. In a typical soret synthesis, the homogenous NP solution is taken in glass vials and subjected to low temperatures (−80 °C, −150 °C and −196 °C) for defined time intervals. The NPs in the solution are stable on account of the electrostatic repulsion between the surface-stabilizing capping groups of NPs. The exposure to low temperature results in the generation of a temperature gradient in the vial. Consequently, the NPs move from the hot end (central region) to the cold end (peripheral region). Eventually, in such adiabatic cooling conditions, the temperature gradient overcomes the electrostatic repulsion, thereby resulting in the generation of stable nano-assemblies. The number of NPs constituting the nano-assembly is directly proportional to the time of exposure to the adiabatic cooling environment. Furthermore, the frozen vials are removed from the cooling chambers and left undisturbed at room temperature for thawing. A clear phase separation is observed upon the formation of sorets. The sorets, which are nano-assemblies, generally separate out into a lower layer due to higher relative mass as compared to the pristine NPs. They are separated and concentrated by centrifugation and utilized for further characterization and plasmonics explorations. It has been noted that the time required for obtaining such nano-assemblies can be significantly reduced by using lower temperatures during the synthesis. Figure 2a presents the number of NPs (Ag and Au) constituting a single assembly obtained by subjecting the NPs solution to −18 °C temperature, for increasing time intervals (60, 90 and 120 min) of adiabatic cooling. It is observed that, with increasing time of adiabatic cooling, the NPs constituting the assembly increase, presenting a higher number of plasmonic hotspots suitable for biosensing applications. Likewise, the experiments with −80, −150 and −196 °C yielded similar trends in the NPs per assembly (increasing with the increase in cooling time) in the case of both the silver and gold cryosorets (Figure 2b,c). 

Importantly, the time required for the formation of nano-assemblies has been drastically reduced from 2 h (−18 °C) to 3 min (−196 °C) for the complete formation of nano-assemblies. Moreover, this technique has also been adopted for the generation of hybrid cryosorets in combination with LRI silica, HRI titania nanospheres and nanorods, as well as GO variants [54]. Since the Ag-TiO_2_ (nanorods) cryosorets [AgTiO_2_(R) CS] yielded the maximum optical coupling efficiency with the underlying GO layer (pre-coated on the metal thin film) and concomitantly augmented SPCE enhancements, this variant has been utilized for the sensing of SPCE reporter molecule (rhodamine B) in drinking water samples; the results are presented in Figure 2d–g. On account of the high >1300-fold SPCE enhancements realized with the AgTiO_2_(R) CS, the device presented high sensitivity with a limit of detection of zeptomolar concentrations (single-molecular limit). The integrated and abundant metal-dielectric hybrid resonances in the AgTiO_2_(R) CS, as well as well their synergistic interaction with the graphene π-plasmons and SPPs of the metallic thin film, assisted in the multiplicative effect of the emitted photon, thereby resulting in high sensitivity [54]. Furthermore, in line with the description provided in earlier sections, the smartphone-based detection system was utilized for achieving the single molecule limit of detection, and the associated luminosity values and the shade cards are presented in Figure 2d–g. Such a robust and reliable smartphone-based detection system assists in cost-effective and portable analyte quantification routes, especially of relevance to medical and environmental healthcare in low-and-middle-income countries. Furthermore, in order to re-emphasize the utility of cryosoret nano-engineering for the development of biosensing frameworks, a comprehensive analysis of the literature is presented in Table 1, where representative examples from the past 10 years (2010–2022) have been tabulated. Here, the performance of different techniques for the detection of rhodamine B (SPCE reporter molecule) and its comparison with the capability of the cryosorets is tabulated, where we observe that the cryosorets render better limit of detection.

Furthermore, it is instructive to comment upon the futuristic scope and opportunities provided by CSNE technology. From a broad perspective, self-assembly is the undercurrent that sustains all forms of living beings on Earth and in aquatic systems. This is simply true on account of the well-known functionality of living cells constituting DNA and RNA, with active biophysicochemical connections in living systems with an inherent memory of self-assembly in them. The ideologies of the self-governing forces in living systems can be extrapolated to nanoconstructs at meso-, macro- as well as nano-scale dimensions using the CSNE technology to obtain materials with tunable optoelectronic properties, amenable for applications in robotics and medicine [130]. While Figure 2i presents the research outcomes that are currently explored with the CSNE, Figure 2j captures the possibilities for future research in this direction, where metal and dielectric nanomaterials can be efficiently integrated in to self-assemblies using adiabatic cooling technology. This can be achieved by implementing anisotropic metallo-dielectric nanomaterials (such as nanostars, nanocubes, nanorods, nanotriangles, nanopipettes and spiky nanourchines, to name a few) with sharp edges that intrinsically sustain tip-to-core plasmon resonances [131,132,133,134], while their cryosorets can support extremely intensified Bragg and Mie plasmons, thereby entering the realm of strong optical coupling. While the understanding of the several opportunities presented by the CSNE aids scientists in developing assemblies with desirable bulk properties, the technology also encourages chemists to incorporate the ligand-exchange methodologies with suitable surfactants and solvents (such as hexane, cetyltrimethylammonium bromide, thiolated polyethylene glycol) to generate mutually ordered and higher ordered super lattice structures. Earlier research on the nanofocus and generation of the so-called ‘hottest hotspots’ has presented an interesting understanding of increases in the EM field enhancements from 580 to 1023 to 2500 for trimers, to pentamers, to hexamers in nano-assemblies, generated on account of the increasing number of plasmonic hotspots (a multiplicative enhancement effect) [135,136]. Consequently, the emplacement of cryosorets over the metal-dielectric-metal (MDM) substrates and such analogous platforms is expected to yield intriguing phenomena. In this context, the CSNE presented a key technology, which not only yield nano-assemblies to generate multiple hotspots with first, second, third and fourth generations of hotspots, but also assisted in the formation of lower and higher order nano-assemblies with robust design principles for potentially economical large-scale production and utilization in multi-disciplinary research, including plasmonics, antimicrobial, anticancer, antiviral, solar cells, smart textiles, robotics and warfare instruments design, to name a few.

## 4. Ferroplasmon-on-Mirror (FPoM) Technology

Fundamentally, the ferromagnetic nanoparticles that are separated by a small nanogap between them assist in the formation of the gap-based nanoantenna effect and exhibit light-matter interactions in the realm of ‘extreme nano-optics’ [137]. This is on account of the high EM field intensities generated in the nano-junctions of the NP-pair-oriented assembly of NPs, as well as random aggregates, that have substantial influence on the adjacently located radiating dipoles. One such example of nano-assembly formation with cryosorets is discussed in the preceding section, albeit without the observation of magnetic dipole resonances. Initially, plasmonic hotspots were studied and pioneered by Li and co-workers [138] in self-similar arrays of nanomaterials. Following this understanding, several explorations have been reported and expansively discussed in a variety of reviews [139,140,141,142]. Such optical nano-antennas that sustain the gap-antenna effect favor the active conversion of freely propagating radiation, also known as free space radiation, into the localized optical density of states, and vice versa. In light of these advantages offered by materials sustaining nanogaps, great interest has been focused on generating controllable and reproducible (programmable) hotspots. However, it has been observed that although theoretical understanding demonstrates intriguing plasmonic effects, the feasibility of fabricating nano-assemblies with programmable hotspots has remained a daunting challenge, especially while magnetic NPs are incorporated into the synthesis. 

In this regard, as an alternative approach to generating effective plasmonic hotspots, different nanomaterial systems in core-shell and decorated architectures have been studied in the well-known nanoparticle-on-mirror (NPoM) configuration [143,144,145]. The NPoM configuration assists in the formation of highly focused optical fields in the localized cavities (with high Purcell factor: low mode volume and high temporal component) within the nanogaps created between the ferromagnetic NP and the metallic thin film (mirror) [146,147]. From a photoplasmonics perspective, the emplacement of the ferromagnetic NP over the metallic thin film results in symmetry breaking at the interface, and the NPs couple with its mirror image in the metal thin film, thereby resulting in the essential hybridization of magnetic plasmons from the ferromagnetic NPs and the SPPs of metal thin film. 

The fundamental physics principles of the optical coupling between the plasmonic NPs and the metallic thin film, as well the ferromagnetic NP (sustaining magnetic dipole resonances) and the metallic thin film, have been discussed in different reviews [148,149], especially from the magneto-plasmonics perspective. Counterintuitively, while the NPoM and associated research have developed as a mature sub-field of nanophotonics, the recent synergy of radiating dipoles (or fluorescent moieties) with NPoM has resulted in the origin of the latest SPCE explorations. Basically, the broad classification of magneto-plasmonics involves the study of materials, which demonstrate a magnetic dipole response (like that of a ferromagnetic nanomaterial) and its interaction with plasmonic nanomaterials or metal thin films. However, ferroplasmonics is a sub-field of plasmonics, which demands the study of nanomaterials that are intrinsically ferromagnetic, as well as plasmonic. In other words, the prerequisite for a nanomaterial to be considered a ferroplasmonic material consists of the material sustaining a ferromagnetic response, as well as a LSPR that is evidently present in spite of the strong magnetic response of the nanomaterial. Although, such ferroplasmons are synthesized and explored for different applications [146,147], the focus in this review is gathered on the utility of such ferroplasmonic NPs over the SPCE platform for the generation of electric as well as magnetic flux modes sustaining associated hotspots. In a recent work [71], this was accomplished with the careful engineering of diamagnetic metal (Ag) and paramagnetic non-metal (Nd_2_O_3_ NRs) as starting materials to generate a ‘plasmon glue’ with the ability to fuse different low dimensional nanosystems (such as carbon dots and graphene oxide). It is instructive to discuss the mechanisms of formation and functionality of ferroplasmonic NPs. A nanomaterial that presents a ferromagnetic response in addition to a strong surface plasmon band in the absorbance spectra is categorized as a ferroplasmonic NP [71]. Synthesis is achieved using a facial, rapid and cost-effective approach. A simple mixture of the Nd_2_O_3_ nanorods and Ag^+^ ions is exposed to UV irradiation. The mechanism of NdAg formation proceeds through a photo-induced electron transfer mechanism where the Nd_2_O_3_ nanorods (high energy radiation absorbers) instantaneously reduce the metal ions to respective zerovalent species, thereby initiating the nucleation process. Further details regarding the functionality of natural organic matter (NOM) and surface-adsorbed carbonates as effective chemical moieties for hastening the NPs synthesis and reaction propagation is detailed in earlier work [71]. The process is similar to earlier work on the generation of Ag-CD nanohybrids, where a direct exposure of CDs and Ag^+^ ions to UV light results in the generation of AgNPs that decorate over the CDs. However, the Ag-CD nanohybrids lack the ferromagnetic response, thereby requiring the use of other dielectric materials that satisfy the prerequisites of ferroplasmonic NPs. The NdAg nanohybrids thus formed are centrifuged and separated from unreacted precursors, and are further utilized for characterization and plasmonic explorations. Interfacing such ferroplasmonic NPs over the metallic thin film assists in the generation of electric as well as magnetic hotspots. While the FDTD simulations that theoretically validate the generation of such hotspots have been elaborately presented in earlier work [71], this focus review presents the key highlights of the developed technologies for applications in biosensing.

**Figure 3:**
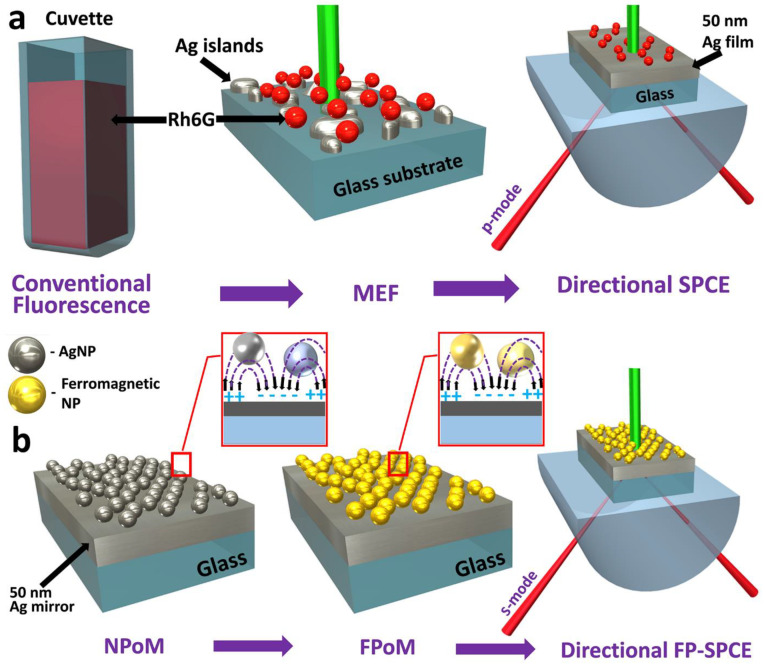
(**a**) Progress in conventional fluorescence-based detection techniques towards metal-enhanced fluorescence (MEF) and directional surface plasmon-coupled emission (SPCE) technology using metallic thin films. (**b**) Progress in Nanoparticle-on-mirror (NPoM) achieved towards Ferroplasmon-on-mirror (FPOM) to realize Ferroplasmon-coupled SPCE technology. Interestingly, the polarization of the out-coupled emission can be tuned from p-polarized to s-polarized by changing the nanointerface from spacer to cavity for the same material, with the use of ferroplasmons Adapted with permission from [71].

The hybrid ferroplasmon of dielectric Nd_2_O_3_ NRs and plasmonic Ag (abbreviated as NdAg) resulted in the accomplishment of room-temperature ferromagnetism (RTFM), while the LSPR mode sustained by the Ag (constituting the NdAg hybrid) was not hampered. Such ferroplasmon NdAg hybrids, while interfaced over the SPPs of metal thin film with SPCE reporter molecule (RhB), have assisted in the formation of a striking polarization switch towards s-polarized emission while studied in cavity nanointerface vis-à-vis a spacer interface where the conventional p-polarized emission was observed [71]. Figure 3a presents a schematic understanding of the latest developments in fluorescence spectroscopy, where the study of radiating dipoles with plasmonic NPs (such as Ag chosen for representation in Figure 3a) has assisted in enhancing the radiative decay rate and photostability, notwithstanding the limitation of isotropic emission in PEF. The utility of prism coupling technology has aided in circumventing this drawback, as observed in the SPCE platform that is exceedingly directional and highly p-polarized (on account of the phase matching conditions satisfied at the metal-dielectric nanointerface, which can be derived from Maxwell’s equations).

While the general observations of the SPCE platform hitherto have been constrained to the use of p-polarized emission, the utility of s-polarized emission has remained unexplored. The conditions for observation of s-modes in SPCE technology demand modulation in the thickness of the dielectric nanolayer, as a consequence of which multiple modes constituting s-modes are generated. Therefore, although such attempts are successful in the generation of perceivable s-modes, the overall collection efficiency of the underlying SPCE substrate is routinely hindered, demanding the use of additional optical components (such as focusing lenses and conical mirrors) to avoid such loss [150,151]. From this perspective, nano-engineering materials that sustain magnetic flux modes when interfaced over the metallic thin films are conducive for the realization of s-modes without loss in collection efficiency. Figure 3b captures the executive summary of such observations made with the use of ferroplasmonic NPs that display a ferromagnetic response and concomitant s-polarized emission intensities when studied in the cavity nanointerface. The p-polarized emission was implemented for sensing 4-nitrophenol (LOD: 100 fM) in the conventional SPCE platform. Furthermore, the s-polarized emission was utilized for sensing allura red (LOD: 10 aM) in drinking water samples, thereby demonstrating the robustness and high throughput for ultra-sensitive quantitative analyte determination. Furthermore, in order to emphasize the utility of FPoM for the development of biosensing frameworks, a comprehensive analysis is presented in Table 2. Here, representative examples from the past ~10 years (2010–2022) have been considered for comparison. The performance of different techniques for the detection of the allura red and its comparison with the FPoM platform is tabulated. We observe that the FPoM platform renders an enhanced LOD and linear sensing range, in addition to enabling the smartphone-based detection platform (discussed in detail in earlier work [71]). Moreover, the synthesized ferroplasmonic NPs exhibited tunable structural, morphological, optical and magnetic properties, thereby enabling their usage in inter-disciplinary applications. Consequently, the metal-dielectric nanohybrids not only exhibited a high sensing performance in the SPCE platform, but also performed as excellent candidates for antimicrobial (antibacterial and antifungal) as well as anticancer applications [71]. 

## 5. Photonic Crystal-Coupled Emission (PCCE) Technology

**Figure 4:**
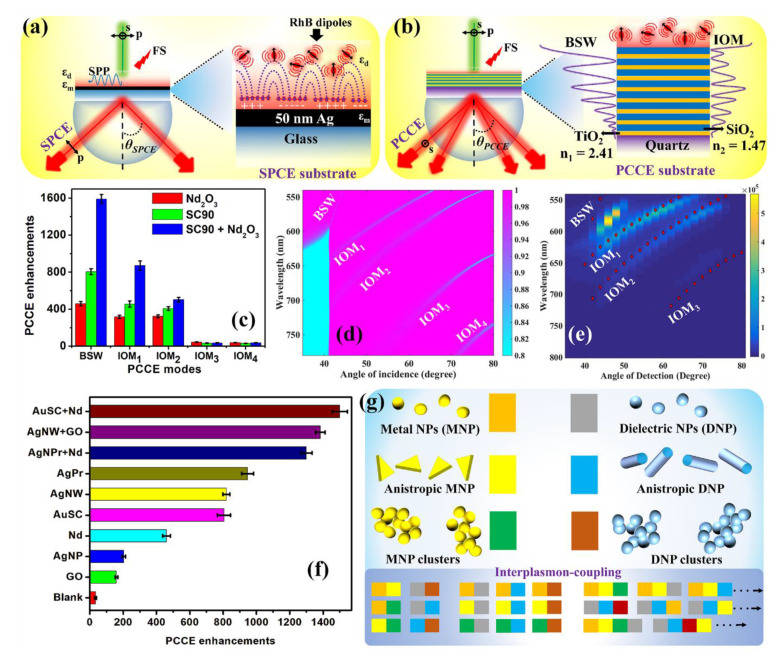
(**a**) Conceptual schematic of coupling of emission with (**a**) propagating surface plasmon polaritons (SPPs) of metal thin film and (**b**) surface-trapped modes of 1DPhC. The magnified images on the right side of (**a**,**b**) display the coupling at the nanointerface (metal-dielectric and 1DPhC-dielectric interface). (**c**) PCCE enhancements for different modes (BSWs, IOM_1_, IOM_2_, IOM_3_ and IOM_4_) for Nd_2_O_3_, SC90 and SC90 + Nd_2_O_3_ samples. (**d**) Numerical dispersion diagram in the angular range from 35° to 80°. The color bar represents the reflectance in arbitrary units. (**e**) Dispersion diagram presenting overlap of numerical (dotted line) and experimental data for 60 nm PVA-doped SC90 + Nd_2_O_3_ in the PCCE platform. We have chosen this data for the representation of typical emission patterns and theoretical understanding observed using the PCCE platform. (**f**) Experimental fluorescence enhancements obtained with different nanomaterials in the PCCE platform. (Acronyms: GO: Graphene oxide; AuSC: gold soret colloids; AgNWs: silver nanowires; AgNPrs: silver nanoprisms; Nd/Nd_2_O_3_: neodymium (III) oxide). (**g**) A snapshot of the futuristic scope of the photoplasmonic nanointerface indicating different opportunities of combining plasmonic and dielectric materials in SPCE, FPoM and PCCE platforms. The color shades are used to make the representation much simpler. Adapted with permission from [11].

Although the SPCE platform has emerged as a versatile biosensing technique, it suffers from inevitable Ohmic losses, as well as surface instability, thereby making the process difficult for extended bioconjugation and related biosensing applications. Ohmic losses result in surface-induced quenching, while the radiating dipoles are in close vicinity (<5 nm), thereby compromising the expected EM field enhancements in the SPCE framework [167,168,169]. In this regard, there has been research conducted to explore materials with less loss and, thanks to dielectric materials, they provide alternative platforms in the form of photonic crystals, opals and inverse opals [170,171,172,173,174,175,176]. Although diverse types of photonic crystals have been explored for interdisciplinary applications, this focus review aims to provide a glimpse of the scientific progress from the perspective of SPCE technology. In 2020, Ramamurthy and Bhakta et al. developed an analogous platform to that of SPCE using alternating layers of high and low refractive index materials, which are generally known as photonic crystals [40,42]. While the surface plasmons are excited in the process conditions of SPCE (Figure 4a), the surface electromagnetic waves, termed as Bloch surface waves (BSWs) and internal optical modes (IOMs), are excited in the processes involving the one-dimensional photonic crystal (1DPhC) [11,40,42] under appropriate optical excitation conditions. As the emission from the fluorophores situated at the surface of the 1DPhC can be optically coupled to the BSWs and IOMs sustained by the 1DPhC in a simple RK configuration, the out-coupled emission from the prism side is termed as photonic crystal-coupled emission (PCCE). As shown in Figure 4b, the 1DPhC discussed in this focused review is comprised of alternating layers of HRI (TiO_2_, thickness: 55 ± 5 nm, RI: 2.41) and LRI (SiO_2_, thickness: 105 ± 5 nm, RI: 1.47) quarter waveplate nanolayers. 

The details of the fabrication procedure and the associated technologies are presented in detail in related earlier works [11,40,73,74]. The characteristic feature of the 1DPhC enabling its functionality for effective biosensing stems from its photonic band gap (PBG) property. PBG is the region of the EM spectrum for which certain frequencies are not allowed to pass through the 1DPhC. Consequently, for the radiating dipoles that emit radiation in the region of PBG, the emission is completely reflected into the incident medium. While the 1DPhC coated with the radiating dipoles is coupled to a prism and excited in RK optical configuration, the emission is observed for specific angles where the phase matching conditions are satisfied. Such domains of specific angles and frequencies where the emission is observed are on account of the ability of the 1DPhC to sustain (i) Bloch surface waves and (ii) internal optical modes. While the BSWs are surface EM waves presenting high field intensity at the surface of the 1DPhC, the IOMs are photonic mode densities that are sustained within the 1DPhC. The effect of the number of different bilayers constituting the 1DPhC, the thickness of the dielectric nanolayer over the 1DPhC as well as the effect of different laser sources in modulating the response of BSWs and IOMs have been captured in detail in earlier works in this domain [11,40,73,74].

The representative PCCE enhancements for different modes (BSWs, IOM_1_, IOM_2_, IOM_3_ and IOM_4_) obtained using different nanomaterials such as Nd_2_O_3_, SC90 and SC90 + Nd_2_O_3_ samples have been presented in Figure 4c. It is observed that the PCCE enhancements were maximum for the BSW-coupled emission, followed by different IOMs [11]. This is because the BSWs occur very close to the critical angle, as observed in Figure 4b (theoretical dispersion diagram of the 1DPhC with a 60 nm PVA overcoat acting as a dielectric), in addition to being a surface EM wave vis-à-vis the IOMs that are modes sustained within the 1DPhC. Essentially, this means that the probability of finding a photon is significantly higher in these regions of the dispersion diagram, where a sharp dip in reflectance is observed. In other words, the photonic mode density for regions of specific wavelengths and angles is at maximum, assisting in higher fluorescence enhancements [11]. Extensive simulations based on MATLAB coding, the transverse matrix method and the associated electric field intensities for 1DPhC constituting different number of bilayers of TiO_2_ and SiO_2_ have been discussed in earlier works, and interested readers are directed to associated references [11,40,73,74]. The dispersion diagram presenting the overlap of numerical (dotted line) and experimental data for 60 nm PVA-doped SC90 + Nd_2_O_3_ in the PCCE platform is shown in Figure 4e. From Figure 4d,e, two important observations can be made. Firstly, there is an excellent co-relation of the modes obtained between the numerically analyzed dispersion diagram and the dispersion diagram experimentally obtained. Secondly, an effective comparison of the dip in the reflectance of Figure 4d and the pattern of fluorescence intensity observed in Figure 3e clearly indicates that a good correspondence can be drawn between the strength of the expected mode and the observed intensity variations in the PCCE spectra. In summary, it is important to note that the PCCE platform equips the photonics experimentalists to tap the BSWs and the desirable IOMs based on the needs of the experiment. This also presents opportunities for multi-analyte sensing, especially while working with molecules in a matrix that can couple with radiating dipoles emitting at different regions of the EM spectrum. Consequently, the associated modes in the PCCE platform independently couple, thereby enabling multiplexing in analytical chemistry. 

Furthermore, different nanomaterials and nanohybrids (Figure 4f) have been explored in the PCCE platform for the generation of efficient EM hotspots in an attempt to further amplify the fluorescence emission signal. At this juncture, it is worth mentioning that the blank measurements in the PCCE platform rendered a ~40-fold increase in the fluorescence enhancements as compared to the ~10-fold fluorescence enhancements observed in the SPCE platform (Figure 1e) [40]. This is primarily on account of the lossless nature of the PCCE platform as compared to the lossy SPCE platform. In this regard, the fluorescence enhancements with different materials observed in the PCCE platform are significantly higher compared to those observed in the SPCE platform, presenting a significant advancement in biosensing platform development. From Figure 4f, we observe that tunable PCCE enhancements can be obtained as per the desired sensitivity of the proposed biosensor, by utilizing appropriate nanomaterial and/or a nanohybrid. Furthermore, in order to emphasize the importance and relevance of the PCCE platform for the detection of desired analytes in biosensing frameworks, we present a comprehensive analysis in Table 3 considering the detection of a biologically relevant molecule, cholesterol. Research works from the past 10 years are presented with the details of the nanomaterial used, the LOD and the linear range obtained. It is observed that the PCCE platform provides better LOD and a wide linear range for the detection of cholesterol. Furthermore, the futuristic scope of effective nano-engineering using the SPCE, FPoM and PCCE platforms is comprehensively captured in Figure 4g and is elaboratively discussed in the subsequent section, where the highlights of the presented technologies are succinctly captured, presenting a newer outlook and opportunities for further research and development in this direction.

## 6. Futuristic Scope and Opportunities

From Figure 4g, we can observe that the permutations and combinations of the nanoparticles made up of metallic and dielectric materials in terms of size and shape open up several opportunities for studying the novel physicochemical attributes of such hybrid materials. Moreover, interfacial interactions of these isotropic and anisotropic nanomaterials using the SPCE, PCCE and FPoM platforms are anticipated to generate new light matter interactions and are expected to present new insights into the broad arena of nanophotonics and plasmonics. Furthermore, it should not go without mentioning that several 2-dimensional materials (including WS_2_, MoS_2_, WSe_2_, TaS_2_, TaSe_2_ to name a few) have been exponentially explored for applications in nanophotonics on account of their distinct optoelectronic properties [192]. In light of these observations, it is important to note that the nano-assemblies constituted by the plasmonic, dielectric (HRI and LRI) as well as low dimensional nanosubstrates would render tunable properties for desired applications. Such careful engineering of nanomaterials would require an adequate understanding of the chemical and physical properties of the starting materials in order to comprehend the overall performance of the nanohybrid in the expected application. Unfortunately, it is important to note that the discipline-driven explorations in the broad field of nanoscience and nanotechnology have often resulted in the generation of toxic chemicals and side products. From this perspective, in the recent past, focus has shifted towards the development of nanotechnology by considering the principles of green nanochemistry and associated policies [193,194,195,196]. Such scenarios demanding the synthesis of novel nanohybrids, as well as abiding by the principles of green nanochemistry, have been a long-standing challenge to achieve the ‘one-health perspective’. Although extensive theoretical and simulation-based studies can suggest the best combination of potential materials (from the domains of metal, dielectric and 2D materials) for advanced photon-driven processes (such as photonic sensors, photovoltaics, photo-diodes, photocatalysis, photonic lasers, to name a few), such computational methodologies often require implementation of wide-ranging mathematical tools and boundary conditions, with experts for understanding and processing the obtained data. Undoubtedly, although these analyses can yield desirable outcomes to support the experimental results, the potential of the simulations to certainly predict the outcomes of the real-time experiments in laboratory conditions is valid semi-quantitatively as it becomes impractical and irrational to expect to entirely mimic theoretical models in laboratory-confined settings. This is on account of the different approximations and simplifications carried out while performing a theoretical analysis, with several troubleshooting steps.

In light of these observations, it is important to utilize the advantages offered by the advent of the latest tools with regard to artificial intelligence (AI), as it is equipped with the potential to overcome the above-mentioned challenges in designing an appropriate experiment. In this section, the aspects of futuristic scope in this direction are discussed with new insights. As presented in Figure 4g, the possible permutations of combinations of metal, dielectric and 2D materials at nano-dimensions offers several opportunities for advanced research with the generation of a library of novel dimers, trimers, tetramers, pentamers, etc. Firstly, the neural network-based machine learning strategies can be incorporated to accurately predict the functionalities of myriad combinations of materials at nanoscale regime, by feeding the optical, chemical and material characteristics of individual components from the existent dataset (extractable from the relevant publications in the literature). The automated and rapid analyses of the well-trained and devoted neural networks can be performed to recommend the mandatory design parameters (and experimental constraints) for the generation of nanohybrids in order to justify the application needs. Moreover, the screening of such methodologies can be further streamlined to processes involving the usage of biocompatible starting materials in order to comply with the principles of green nanochemistry. The machine-learning algorithms can be tailor-made to categorize such demands from a bionano-inspired perspective, by segregating optical properties of desired materials from the massive set of data points (or procedures) for engineering nanomaterials of specific nano-geometries and properties. Moreover, it is informative to mention the advantages of such approaches from the perspective of the rapid growth of the semiconductor industry and related technologies. For instance, from earlier sections, it has been observed that the developed platforms (SPCE, FPoM and PCCE) can be synergized with the ubiquitously available smartphone-based detection systems, thereby simplifying the process parameters in field trials. In other words, the utility of smartphones by health practitioners and environmentalists aids in obtaining swift feedback from experts, by simply feeding the data (from the real-time collection of samples such as blood or contaminated water/soil) of the disease exposure to the in-built software present in smartphones, and with the appropriate use of cloud computing and AI technologies for data processing, streamlining, storage, transfer and virtual feedback from experts. In summary, it is worth emphasizing that smartphone-based biological and environmental health monitoring would be extremely simplified with the adequate use of AI tools to perform functions such as: (i) the optimization of multifunctional properties needed for a specific application; (ii) streamlining and validating the incorporation of bioinspired nano-architectures; (iii) utilizing the portable smartphone-based detection technologies for point-of-care diagnostics, amenable for common man, in resource-limited settings [64]. With the realization of such goals, the future of medical healthcare is anticipated to be substantially affordable, sensitive, specific, user-friendly, rapid, equipment-free and easily deliverable to the end user (abbreviated as “ASSURED”), which is the underlying research standard for point-of-care (POC) diagnostics as defined by the World Health Organization (WHO) [64]. 

Furthermore, the envisaged applications and potential research directions with the use of ferroplasmons and their cryosoret nano-assemblies necessitate a comment. Conventionally, solid-state mechanochemical reaction techniques have been adopted for the synthesis of hybrid decorated and core-shell nano-architectures, with the use of seed-directed and surfactant-driven nano-chemistry principles. The latest ferroplasmon technology, demonstrated in the year 2023 [71], presents a new opportunity for researchers to explore similar such materials with tunable magnetic and optical properties using other lanthanide oxides and plasmonically active coinage metals. Such explorations aid in obtaining a photo-plasmonic response, as per the requirement, in the desired region of the EM spectrum (be it UV, visible, NIR or IR). The bionano-inspired technologies using the concept of ‘plasmon glue’ may be effectively incorporated to explore cost-efficient and environmentally benign approaches in nanosynthesis. Moreover, the monotonic functionalities in single-component homogeneous nanostructures can be circumvented using plasmonic heterostructures by combining different elements at the nanoscale from different parts of the periodic table, with the use of plasmon glue and cryosoret routes. It is believed that the ‘new-yet-unexpected’ properties of nanohybrids would be possible through such explorations, presenting ‘multi-component nanoparticles’, and thereby significantly simplifying the existing technologies, as the nanohybrids would offer tailorable properties for targeted applications [197]. These studies also present an avenue for the utilization of external electric and magnetic fields to hasten the process of bioconjugation and other related processes (magneto-optics and magneto-catalysis) involving ferroplasmonic nanohybrids [198]. It is well-known that multi-junction device technology for applications in LEDs, photovoltaics and solar cells significantly depends on the electronic band gap of the materials under consideration [199]. As the plasmon glue technology renders effective means for tuning the band gaps of nanohybrids, tunable localization and the densification of charge in nanosolids can be achieved for different applications in nanoelectronics, presenting great scope for further research in this pathway [200]. Additionally, the ferroplasmonic cyrosorets are yet to be explored in different scenarios related to electrospun nanofibers (for channelizing the light waves in the nanofibers), sputtered surfaces (to generate hybrid photo-plasmonic coupling) and in combination with ferroelectric and upconversion nanosystems (for utility in capacitors, ultrafast switches, computers, miniature X-ray and neutron sources) to understand the merits and demerits of the micro-nano technological implications of devices stemming from such studies. In addition, the unexplored attributes of the ferroplasmons generated with lanthanide oxide (f-block elements) and plasmonic metal are the utility of their intrinsic luminescent properties for up- and down-conversion, as well as for photothermal, chemo-photothermal, MRI and CT contrast imaging applications on account of their high absorption coefficient in the NIR region [first (700−950 nm) and second (1000−1400 nm) biological window] [71,201]. Fundamentally, from a materials chemistry point of view, the spin and carrier densities associated with lanthanide oxides have been increasingly investigated for the realization of dilute magnetic semiconductors (DMSs) and dilute magnetic oxides (DMOs). In this regard, semiconductor-metal nanohybrids with ferroplasmonic properties are expected to find relevant applications in spintronic devices. Furthermore, the metallo-dielectric nanohybrids comprised of HRI, LRI as well as metallic components can be systematically studied for their performance as enhancers of SERS signals. Furthermore, adequate supramolecular chemical dynamics in living cells can be explored by tagging the elements of study and related processes with the intrinsically luminescent ferroplasmons, thereby assisting a new paradigm in biosensing and POC diagnostics using in situ and operando-Raman spectroscopy. Furthermore, the extended use of ferroplasmonic nanostructures and envisaged cryosorets in PCCE technology, Tamm structures, Omniferroic substrates and biomimetic functional texture-based platforms are expected to yield novel phenomena in harnessing light-matter interactions at advanced functional interfaces for photon-driven applications [11,30,42,202]. In the broad landscape of photo-driven technologies and IoT, the utility of smartphone-based imaging, analysis and interpretation (also discussed in previous sections for biosensing) are anticipated to drive and guide the associated next-gen opto-electronic know-how, thereby making the related platforms simpler to investigate, comprehend and explore for researchers at undergraduate and postgraduate levels, especially in resource-scarce settings [203,204,205].

Furthermore, it is instructive to discuss the importance of choosing a particular technology over another. In this review, the latest advancements in the three major technologies, (i) cryosorets, (ii) FPoM and (iii) PCCE, are discussed. Fundamentally, the cryosorets and the ferroplasmons differ in the materials and method adopted to obtain the hybrid nanostructures. While the low temperatures drive the cryosoret fabrication, the ferroplasmons are synthesized using a photo-induced electron transfer technique between a metal and a dielectric. Further, while the cryosorets yield precise nano-assemblies, the ferroplasmons are nanohybrids (decorated or core-shell) of metallic and dielectric nanomaterials. Importantly, it has been observed that the cryosorets assist in dequenching the emission from fluorophores on account of the inter-plasmonic coupling between the NPs in the cryosorets. This technology has the potential to be adopted for enhancing the signal intensity of fluorophores in the development of certain bioassays, where low quantum yield fluorophores are inevitably used. Furthermore, the bioconjugation of intrinsically fluorescent proteins and other biomarkers (with low quantum yield) over the cryosorets would render high sensitivity compared to the performance of pristine nanomaterials. Furthermore, biosensing approaches based on conventional ELISA technique can be significantly improved with the use of FPoM technology. Appropriate functionalization of the ferroplasmonic NPs with the secondary antibodies would reduce the time required for completing the bioassays, as the external magnetic field can be use hasten the process of conjugation of the ferroplasmon-tagged secondary antibody on to the FPoM substrate that is pre-coated with a primary antibody and associated antigens. That is, bioassays that rely on the application of an external magnetic field can be significantly improved by the incorporation of ferroplasmonic NPs. Moreover, the ferroplasmons are intrinsically made up of metal-dielectric nanohybrids with tunable optical band gaps, a surface area and structural and magnetic properties. Hence, these nanomaterials not only find application in plasmonic biosensing, but are also expected to be of use in interdisciplinary fields, including antibacterial, anticancer, electrochemical sensors and solar cell platforms. Further, certain assays demand multiple bioconjugation steps, such as wash steps and long incubation time intervals for the development of sensing technologies. In such scenarios, the SPCE platform suffers from surface-induced quenching on account of Ohmic losses, as well as surface chemical instability due to oxidation (especially with the use of Ag thin films as SPCE substrates). In this context, the PCCE platform can be utilized to provide chemically stable substrates with fewer loss attributes. This is because the PCCE platform is fabricated using dielectric nanolayers with the complete elimination of metallic substrates. Moreover, the PCCE substrates enable researchers to use the same platform multiple times after subjecting them to appropriate washing protocols. Furthermore, the limitations and challenges of the three technologies are discussed in the subsequent section.

## 7. Limitations and Challenges

While cryosorets and FPoM and PCCE technologies present several advantages with new opportunities for future research, there are certain limitations and challenges that need to be addressed. Firstly, although cryosoret nano-engineering assists in the rapid formation of precise nano-assemblies, the orientation of the NPs in the synthesized assemblies are anisotropic, as it is a self-assembly process. Consequently, the method renders negligible control over the directed orientation of NPs in self-assembly. In this regard, changing experimental parameters, such as pH and the addition of certain surfactants, might provide some degree of control over directed self-assembly in the generation of cryosorets. Moreover, the feasibility of generating the cryosorets over the chemically functionalized substrate is less understood. Consequently, this remains a major limitation of the protocol associated with the use of low temperatures for the generation of nano-assemblies. The utility of cryosorets and ferroplasmonic NPs for different applications is hampered on account of less information being available regarding their physicochemical properties from a materials chemistry perspective. The real and imaginary components of the dielectric constants, as well as the refractive indices of the synthesized materials, present potential for interdisciplinary applications. For instance, Electron Energy Loss Spectroscopy (EELS) and associated technologies might be utilized to fully characterize the fundamental attributes of cryosorets and ferroplasmonic NPs from a materials chemistry perspective. Currently, the performance of ferroplasmon is demonstrated using lanthanide oxide, Nd_2_O_3_ upon its interaction with plasmonic Ag. In this regard, another important limitation of the ferroplasmonic NPs is the unknown properties of the underexplored metal-dielectric hybrids that could be explored using other lanthanides. Moreover, the lack of ability to decouple the emission from the adjacently located luminophore (such as rhodamine) and the intrinsically fluorescent lanthanide oxide (due to f-f transitions) is a major challenge. In this regard, experiments carried out so far have not utilized the laser excitation source in the absorption maximum of the ferroplasmonic NPs to avoid the contribution from the intrinsic fluorescence of lanthanide oxides. From a plasmonics point of view, it is advisable to utilize the laser sources that are close to the plasma frequency of the metal and as high as possible (in terms of frequency), to lessen the contribution from the imaginary component of the dielectric constant (note: metals are transparent for excitation sources with frequencies higher than the plasma frequency of metal). In this regard, another limitation of the cryosorets and the ferroplasmons is the requirement of adequate technologies to precisely determine the dielectric constant of these nanohybrids.

Although the PCCE substrate presents several advantages compared to the SPCE platform, the fabrication methodology based on the dip-coating method is time and labor intensive, requiring the use of a high-temperature furnace and well-equipped personnel. In this regard, there is a driving need for the development of photonic crystals using cost-effective methods such as spin-coating using high and low refractive index polymer nanolayers. However, such techniques do not yield PCCE platforms with high intensity surface EM waves compared to that obtained using the structurally stable solid surfaces fabricated via TiO_2_ and SiO_2_ nanolayers and similar materials. Additionally, the platforms obtained via polymer nanolayers are prone to degradation while building the sandwich-based ELISA for biosensing applications, and hence, remain a major drawback. Therefore, there exists a trade-off between the desirable high field intensity and the availability of simple fabrication routes to achieve the same. Moreover, slight changes in the surface roughness of the dielectric nanolayer over the 1DPhC, as well as small deviations in the angle of collection, significantly effect the successful observation of the BSWs. Additionally, the methodologies required to fabricate 2D and 3D photonic crystals are highly complex, requiring highly skilled personnel. The large number of bilayers in a 1DPhC assist in the generation of enhanced EM field intensity vis-à-vis a smaller number of bilayers. However, this is accompanied with the shortcoming of restricting the incorporation of smartphone-based detection technologies. This is because of the ability of the large number of bilayers to prevent the easy observation of the out-coupled emission in the PCCE platform via smartphone cameras. On the other hand, a fewer number of bilayers renders a lower sensitivity of the device. From this perspective, newer materials need to be investigated so as to overcome the abovementioned challenges and limitations. A summary of the methodology, LOD, performance in terms of merits and demerits of the conventional fluorescence spectroscopy, SPCE, CSNE, FPoM and PCCE platforms is tabulated in the summary in Table 4. These details are envisaged to assist researchers in the broad domain of SPCE and biosensors to further explore nano-engineering and biosensing modalities in this direction.

## 8. Conclusions

Recently, the broad arena of spectro-plasmonics has been explored with different fluorescent moieties and novel plasmonic interfaces to achieve higher enhancement in emission intensity, with an attempt to augment the sensitivity of related detection technologies. Although the SPCE platform has emerged as a versatile technology for the realization of functional interfaces for investigating different processes and biosensing approaches, the high Ohmic losses and surface instability encountered in them remain an inevitable challenge. Against this background, different nano-geometries have been explored to realize higher SPCE enhancements, in addition to tapping the s-modes, albeit at the expense of complex strategies. In light of these observations, this focused review presents novel nano-engineering methodologies adopted in the recent past to realize unprecedented SPCE enhancements. Furthermore, the alternative biosensing platforms developed with the judicious integration of micro-nano-engineering and materials chemistry approaches have been detailed, providing an overview of cryosoret nano-engineering (CSNE), ferrroplasmon-on-mirror (FPoM) and photonic crytal-coupled emission (PCCE) technologies. In addition to providing a comprehensive analysis of the tunable SPCE and PCCE enhancements with myriad nano-engineering systems, this focus review elaborately deliberates the futuristic scope and research opportunities in the direction of cryosorets, ferroplasmonic resonance glue, FPoM and PCCE platforms. The latest technologies of photon-driven processes concisely captured here are expected to be immediately deployed by researchers and scientists working in the broad area of nanophotonics, plasmonics, materials engineering, POC diagnostics and IoT for achieving enhanced performance of the allied technologies.

## Figures and Tables

**Table 1:** Comparison of the performance of the techniques/strategies developed to detect rhodamine B with the SPCE platform using cryosorets, presented along with their limit of detection (LOD) and the linear sensing range.

Sl. No.	Strategy/Approach Adopted	Nanomaterial Used	Year	LOD	Linear Range	Reference
1	Chromatography	-	2010	1 ng g^−1^	-	[115]
2	Chromatography	-	2011	3.14 μg L^−1^	0.25–3.0 mg L^−1^	[116]
3	Fiber optic-linear array detection spectrophotometry (FO-LADS)	-	2012	1.05 μg L^−1^	5–100 μg L^−1^	[117]
4	Voltammetry(Cyclic voltammetry and differential pulse voltammetry)	-	2013	2.93 μg L^−1^	4.78–956.1 μg L^−1^	[118]
5	Spectrophotometry	Multi-walled carbon nanotubes	2014	1.93 ng mL^−1^	5–450 ng mL^−1^	[119]
6	Surface-enhanced Raman spectroscopy (SERS)	Silver nanoparticles (AgNPs)	2015	10^−6^ g g^−1^	10^−2^ g g^−1^ to 10^−6^ g g^−1^	[120]
7	High-performance liquid chromatography (HPLC)	Polystyrene-coated magnetite nanocomposite	2016	0.0021 µg/mL	1 to 250 µg/L	[121]
8	HPLC	-	2017	2.98 ng mL^−1^	10–1200 ng mL^−1^	[122]
9	Electrochemical method	Carboxylated multi-walled carbon nanotube and ionic liquid modified pencil-graphite electrode (MWCNTs-COOH/IL/PGE)	2018	1 nM	0.005–2.0 2.0–60.0 μM	[123]
10	Electrochemical method	MnO_2_ nanorods/electro-reduced graphene oxide composite modified glassy carbon electrode (MnO_2_NRs-ERGO/GCE)	2019	2.87 μg L^−1^	9.58–479 μg L^−1^; 479–9580 μg L^−1^	[124]
11	Electrochemical method	Multi-walled Carbon Nanotubes Paste Electrode	2020	20.0 nM	0.1–15.0 μM	[125]
12	SERS method	Ag/Au alloy NP	2021	10^−11^ M	10^−11^ to 10^−5^ M	[126]
13	Electrochemical method	Neodymium-based metal-organic framework (Nd-MOF)	2021	3.6 nM	0.08–2.0 and 2.0–40 μM	[127]
14	Electrochemical method	Polyethylenimine and multi-walled carbon nanotubes composite modified glassy carbon electrode (MWCNTs-PEI/GCE)	2022	6.0 nM	0.01−10 μM	[128]
15	SERS method	Au nanoparticles photo-decorated on Cu_2_O microspheres	2022	2.36 × 10^−13^ M	10^−5^ M to 10^−13^ M	[129]
16	Cryosoret nano-engineering (CSNE) in SPCE platform	Silver–TiO_2_ nanorod cryosoret (Ag-TiO_2_ CS) nanohybrid coupling with Ag metallic thin film	2022	1 zM (single molecule detection)	1 zM to 1 pM; 0.01 nM to 1 mM	[54]

**Table 2:** Comparison of the performance of the techniques/strategies developed to detect allura red with the SPCE platform, presented along with their limit of detection (LOD) and the linear sensing range.

Sl. No.	Strategy/Approach Adopted	Nanomaterial Used	Year	LOD	Linear Range	Reference
1	Electrochemical sensor	Multi-wall carbon nanotube film	2010	25 μg L^−1^	50 μg L^−1^ to 0.6 mg L^−1^	[152]
2	Chromatography	-	2011	2.35 μg L^−1^	1–6 mg L^−1^	[153]
3	Chromatography	-	2012	0.17 μg mL^−1^	1–10 μg mL^−1^	[154]
4	HPLC–MS	-	2013	68.02 ng mL^−1^	2.0–100 ng mL^−1^	[155]
5	Triple-wavelength overlapping resonance Rayleigh scattering (TWO-RRS)	-	2014	0.008 μg mL^−1^	0.028–2.48 μg mL^−1^	[156]
6	Electrochemical sensor	Multi-walled carbon nanotubes (MWCNTs) in ionic liquid-graphene oxides	2015	3.0 × 10^−9^ mol L^−1^	5.0 × 10^−9^–4.5 × 10^−7^ mol L^−1^	[157]
7	Electrochemical sensor	Poly (diallyldimethylammonium chloride) functionalized graphene and nickel nanoparticles modified electrode	2016	8.0 nmol L^−1^	0.05–10.0 μmol L^−1^	[158]
8	Fluorescence spectroscopy	-	2017	0.029 μmol L^–1^	0.097–6.0 μmol L^–1^	[159]
9	Electrochemical sensor	Boron-doped diamond electrode	2018	7.0 nmol L^−1^	40.0–770 nmol L^−1^	[160]
10	Electrochemical sensor	carbon paste with cobalt (II, III) oxide composite electrode (CoO_x_/CPE)	2019	0.05 μmol L^−1^	0.10 to 1.00 μmol L^−1^	[161]
11	Electrochemical sensor	Ionic liquid (IL) 1-butyl-3-methylimidazolium tetrafluoroborate and carbon black (CB) nanoparticles	2020	9.1 × 10^−10^ mol L^−1^	3.98 × 10^−8^ to 9.09 × 10^−7^ mol L^−1^	[162]
12	Fluorescence spectroscopy	MoS_2_ quantum dots	2021	1.7 × 10^−6^ M	5.00 × 10^−6^ M to 4.00 × 10^−5^ M	[163]
13	Electrochemical sensor	Raspberry-like In^3+^/NiO hierarchical nanostructure	2021	4.1 nM	0.01–700 µM	[164]
14	Fluorescence spectroscopy	Fluorescence carbon nanodots (FCNs)	2022	23.5 nmol L^−1^	0.01–10.0 μmol L^−1^	[165]
15	Electrochemical sensor	Core-shell Mn_3_O_4_@C nanocubes	2022	0.033 μM	0.1 to 1748.4 μM	[166]
16	FPoM	Nd_2_O_3_-Ag nanohybrids	2023	10 aM	1 mM to 10 aM	[71]

**Table 3:** Comparison of the performance of the techniques/strategies developed to detect cholesterol with the PCCE platform, presented along with the limit of detection (LOD) and the linear sensing range.

Sl. No.	Strategy/Approach Adopted	Nanomaterial Used	Year	LOD	Linear Range	Reference
1	Electrochemical sensor	Carbon nanotubes	2010	10 mg/dL	50–400 mg/dL	[177]
2	Electrochemical sensor	NiO nanoparticles (nNiO, 22 nm)— chitosan (CHIT) film	2011	43.4 mg/dL	10–400 mg/dL	[178]
3	Photoluminescence	CdSe/ZnS quantum dot nanocrystals	2012	0.01 mM	0.01 mM–9.11 mM	[179]
4	Electrochemical sensor	TiO_2_–graphene–Pt–Pd hybridnanocomposites	2013	0.017 μM	5.0 × 10^−8^−5.9 × 10^−4^ M	[180]
5	Electrochemical sensor	Zinc oxide nanotube (ZNT) arrays	2014	0.5 nM	1.0 μM to 13.0 mM	[181]
6	Electrochemical sensor	β-cyclodextrin functionalized graphene	2015	1 µM	1–100 µM	[182]
7	Fluorescent Ratiometric Assay	Semiconducting Polymer Dots	2016	4.9 nM	25 to 350 nM	[183]
8	Electrochemical sensor	Dopamine@graphene (DGr) and bioinspired Au microflowers	2017	3.3 × 10^−19^ M	10^−18^ and 10^−13^ M	[184]
9	Electrochemical sensor	Functionalized nitrogen-doped graphene quantum dots (N-GQD)	2018	80 nM	0.5–100 μM	[185]
10	Colorimetric enzymatic assay	Functionalized silver nanoparticles (AgNPs)	2019	0.014 nM	10–250 nM	[186]
11	Colorimetric sensor	Polypyrrole nanoparticles (PPy NPs)	2020	3.5 μM	10–100 μM	[187]
12	Photoluminescence	CuInS_2_@ZnS (CIS@ZnS) core-shell quantum dots (QDs)	2021	10 nM	0 to 120 nM	[188]
13	Electrochemical sensor	MXene (Ti_3_C_2_Tx)	2021	0.11 nM	0.3 to 4.5 nM	[189]
14	Fluorescence-based sensor	Metal-organic frameworks (MOFs)-doped by europium	2022	0.0105 mM	2 mM to 8 mM	[190]
15	Electrochemical sensor	Multi-walled carbon nanotube (MWCNTs)	2022	3.2 nM	10 nM–75 µM and 100 µM–8 mM	[191]
16	PCCE	Ag nanowires and graphene oxide hybrid coupling over 1DPhC	2023	10 zM	1 zM to 0.1 pM and 0.1 pM to 1 mM	[73]

**Table 4:** Summary table of the comparison of the performance of the recent techniques/strategies developed for biosensing with the SPCE platform, presented along with their limit of detection (LOD), advantages and disadvantages.

Sl. No.	Strategy/Approach Adopted	Methodology in Brief	Advantages	Disadvantages	LOD (Depends on the Quantum Yield of the Radiating Dipole Used)	Ref.
1	Fluorescence Spectroscopy	The emission from the fluorescent molecules present in the cuvette is captured using the detector placed at 90° to the light source	High sensitivity compared to absorbance and other optical methods. Highly versatile method where different biomarkers and analytes (ions and molecules) can be analyzed.	High background noise Low spectral resolution Low signal collection efficiency Emission is not polarized Observation of experimental artifacts in quenching-dequenching studies	Micromolar to picomolar LOD	[10,27,206]
2	Surface plasmon coupled emission (SPCE)	Prism-coupling technique where metallic thin film is coupled to the prism and the emission is monitored via a filter, a polarizer, an optic fiber and a detector	High surface EM field intensity on account of generation of SPPs Low background noise High signal collection efficiency due to sharp directionality of emission Highly p-polarized emission Easy for functionalization studies on the metallic surface	Multiple uses of the same substrate are not possible High Ohmic losses High surface-induced quenching effects observed S-polarized emission is not observed (with the thickness of dielectric <70 nm) The fluorescence enhancements achieved are moderate, ~10-fold as compared to conventional fluorescence technique	Micromolar to picomolar LOD without the use of AgNPs	[8,28]
3	Nano-engineering in SPCE using pristine AgNPs	APTES functionalized SPCE substrate coated with citrate-stabilized AgNPs	High surface EM field intensity with hybrid coupling of LSPR of AgNPs and the SPPs of metallic thin film Low background noise High signal collection efficiency and p-polarized emission Ease of functionalization High fluorescence enhancements (~60-fold) compared to conventional SPCE (~10-fold)	Does not enable exploration of electric and magnetic plasmonic hotspots S-polarized emission is not observed (with thickness of dielectric <70 nm) The fluorescence enhancements achieved needs improvement for enhancing the LOD of analytes	Nanomolar to Picomolar LOD	[32,48]
4	Cryosoret nano-engineering (CSNE)	Adiabatic cooling at low temperatures (−80 °C, −150 °C and −196 °C), thawing, centrifugation and purification	High surface EM field intensity with hybrid coupling of Mie and Bragg plasmons of cryosorets with the SPPs of metallic thin film Low background noise, high signal collection efficiency and highly p-polarized emission High fluorescence enhancements (~1350-fold) compared to conventional SPCE (~10-fold) and SPCE with AgNPs (~60-fold)	Does not enable exploration of electric and magnetic plasmonic hotspots S-polarized emission is not observed (with thickness of dielectric <70 nm) Requirement of adiabatic cooling chambers Generation of anisotropic nano-assemblies Negligible control over directed synthesis as it is a self-assembly technique	Attomolar LOD to Single molecule detection	[54]
5	Ferroplasmon-on-mirror (FPoM)	Sunlight-driven photon-induced electron transfer	Enables exploration of electric and magnetic plasmonic hotspots Enables the observation of p- as well as s-polarized emission (with the thickness of dielectric <70 nm) Low background noise High signal collection efficiency	Requires the use of UV-light for synthesis in the absence of adequate sunlight Necessitates careful engineering of metallic and dielectric NPs to observe ferromagnetic response without damping the LSPR of metallic NPs. Requires the use of high refractive index NPs for effective generation of magnetic plasmonic hotspots	Femtomolar -Attomolar LOD	[71]
6	Photonic Crystal-Coupled Emission (PCCE)	Sol-gel synthesis method coupled with dip coating technique	High surface EM field intensity Low background noise High signal collection efficiency High sensitivity High surface stability Enables multiple uses of the same substrate Negligible surface-induced quenching Enables multiplexed detection of analytes on account of multiple wavelengths coupling at different angles High fluorescence enhancements (~40-fold) as compared to conventional SPCE (~10-fold) in blank measurements without the use of nano-engineering	Difficulty in fabrication of the 1DPhC substrate (with the adequate use of high temperatures and chemical precursors in addition to being time-intensive) Large dependence of dielectric thickness on the BSW coupling, thereby decreasing the PCCE enhancements for slight variations in surface roughness and angle of collection Necessitates careful nano-engineering to enhance the blank PCCE enhancements (40-fold).	Attomolar LOD to Single molecule detection	[42,73]

## Abbreviations

SPCE, Surface plasmon coupled emission; PCCE, photonic crystal-coupled emission; FPoM, ferroplasmon-on-mirror; NPoM, nanoparticle-on-mirror; AgNW, silver nanowire; RhB, rhodamine B; GO, graphene oxide; 1DPhC, 1-dimensional photonic crystal; MEF, metal enhanced fluorescence; BSW, Bloch surface waves; IOMs, internal optical modes; RK, reverse Kretchmann; LSPR, localized surface plasmon resonance; AgTiO_2_ CS, silver titanium dioxide cryosoret; AgAu, silver-gold nanohybrid; AgCD, silver NP decorated-carbon dots; AgNPrs, silver nanoprisms; AgNW, silver nanowire; DNA/Ag/CD, DNA based AgCD composite; Nd_2_O_3_, neodymium (III) oxide; Ag/SLGO, silver NPs decorated on single layer graphene oxide; Ag/lignin, lignin-based AgNPs; Ag/ CNT, carbon nanotubes decorated with AgNPs; AuNS, gold nanostars; Au/SiO_2_, gold NPs decorated on silica NPs; TiCN, titanium carbonitride; C60, carbon allotrope or buckminsterfullerene, (C60-Ih) [5, 6] fullerene.
